# Maternal Gut Microbiome Decelerates Fetal Endochondral Bone Formation by Inducing Inflammatory Reaction

**DOI:** 10.3390/microorganisms10051000

**Published:** 2022-05-10

**Authors:** Yoko Uchida-Fukuhara, Takako Hattori, Shanqi Fu, Sei Kondo, Miho Kuwahara, Daiki Fukuhara, Md Monirul Islam, Kota Kataoka, Daisuke Ekuni, Satoshi Kubota, Manabu Morita, Mika Iikegame, Hirohiko Okamura

**Affiliations:** 1 Department of Oral Morphology, Faculty of Medicine, Dentistry and Pharmaceutical Sciences, Okayama University, Okayama 700-8525, Japan; ikegame@md.okayama-u.ac.jp (M.I.); hiro-okamura@okayama-u.ac.jp (H.O.); 2 Department of Biochemistry and Molecular Dentistry, Faculty of Medicine, Dentistry and Pharmaceutical Sciences, Okayama University, Okayama 700-8525, Japan; hattorit@md.okayama-u.ac.jp (T.H.); fushanqi@s.okayama-u.ac.jp (S.F.); de421016@s.okayama-u.ac.jp (S.K.); kuwahara.miho@s.okayama-u.ac.jp (M.K.); kubota1@md.okayama-u.ac.jp (S.K.); 3 Department of Preventive Dentistry, Okayama University Hospital, Okayama 700-0914, Japan; de20041@s.okayama-u.ac.jp; 4 Department of Preventive Dentistry, Faculty of Medicine, Dentistry and Pharmaceutical Sciences, Okayama University, Okayama 700-8558, Japan; p3a99o50@s.okayama-u.ac.jp (M.M.I.); de18017@s.okayama-u.ac.jp (K.K.); dekuni7@md.okayama-u.ac.jp (D.E.); mmorita@md.okayama-u.ac.jp (M.M.)

**Keywords:** maternal microbiome, endochondral ossification, fetal chondrocytes

## Abstract

To investigate the effect of the maternal gut microbiome on fetal endochondral bone formation, fetuses at embryonic day 18 were obtained from germ-free (GF) and specific-pathogen-free (SPF) pregnant mothers. Skeletal preparation of the fetuses’ whole bodies did not show significant morphological alterations; however, micro-CT analysis of the tibiae showed a lower bone volume fraction in the SPF tibia. Primary cultured chondrocytes from fetal SPF rib cages showed a lower cell proliferation and lower accumulation of the extracellular matrix. RNA-sequencing analysis showed the induction of inflammation-associated genes such as the interleukin (IL) 17 receptor, IL 6, and immune-response genes in SPF chondrocytes. These data indicate that the maternal gut microbiome in SPF mice affects fetal embryonic endochondral ossification, possibly by changing the expression of genes related to inflammation and the immune response in fetal cartilage. The gut microbiome may modify endochondral ossification in the fetal chondrocytes passing through the placenta.

## 1. Introduction

The gut microbiome living in our body has recently been considered to act as one of our organs [1,2]. As well as being involved in the maintenance of homeostatic processes in the body, disturbances in the gut microbiome are closely linked to inflammatory diseases [3,4,5]. Furthermore, the gut microbiome can manage our health and even our behavior [6,7].

In recent years, the role of the gut microbiome in a single individual and the influence of the intestinal microbiome over subsequent generations, such as in the maternal gut microbiome, has attracted much attention. The composition of the maternal gut microbiome changes during pregnancy [8,9,10], and the gut microbiome of pregnant women with gestational diabetes is more diverse than that of normal pregnant women [11]. Moreover, analyzing the mechanistic interactions between the perinatal microbiome, immune system, and nervous system has been continuously intensified [12]. Under germ-free (GF) conditions or antibiotic treatment during fetal or postnatal periods, the development of immune tissue and the expression of inflammatory factors in the nervous tissue are affected [13,14], suggesting that the maternal gut microbiome has a significant influence on the immunological and neurological development of the fetus. The effect of the maternal gut microbiome in children after birth has also been suggested. Maternal dietary intake has also been reported to reduce asthma incidence in children [15,16]. In addition, using a high-fat model of GF mice, the maternal gut microbiome was found to affect future metabolic functions in the offspring [17]. These findings suggest that the maternal gut microbiome has an impact, via the placenta, on the immune and metabolic function building of the offspring.

The gut microbiome is also known to be associated with bone homeostasis and bone disease. The first report in 2012 showed that osteoclast differentiation is inhibited and bone mass is increased under GF conditions [18]. Furthermore, it has been suggested that short-chain fatty acids secreted by the gut microbiome may contribute to osteogenesis by inhibiting osteoclast activity and increasing osteoblast activity [19,20]. Our group has also reported an association between the gut microbiome and bone formation [21]; we found that the microbiome enhances both osteoclast and osteoblast activities by changing expression levels of transcription factors such as Forkhead box g1 and Gata-binding protein 3. As mentioned above, it is becoming clear that the presence or absence of the maternal gut microbiome can affect fetal immunity and nerve tissue; however, most of the reports on the relationship between the gut microbiome and bone development have focused on a single generation [22,23].

In the present study, we focused on the effects of the maternal gut microbiome on fetal bone growth during the embryonic period, particularly endochondral bone formation, by analyzing embryonic cartilage in the presence or absence of the maternal gut microbiome and compared our results with previous reports obtained with adult mice.

## 2. Materials and Methods

### 2.1. Animals

Fetuses at embryonic day 18 from pregnant germ-free (GF) and specific-pathogen-free (SPF) IQI/Jic mice were obtained from the Central Institute of Experimental Animals (CIEA, Kanagawa, Japan). The mice were bred and maintained at the CIEA. The conditions for breeding mice have been described previously [21]. Pregnant GF and SPF female mice were euthanized at 18 days of age by cervical luxation. Thirteen GF and twelve SPF embryos were collected with placentas by cesarean section under sterile conditions and transported to Okayama University. The fetus and placentas were carefully separated, removing extra blood vessels. The rearing of the mice was conducted according to the institutional rules following approval from the Animal Experiment Committee of the Central Institute for Experimental Animals in Japan (18069A).

### 2.2. Skeletal Preparation

Embryos were fixed overnight in 95% ethanol after removing all the skin and internal organs for 24 h at 4 °C. Cartilaginous parts were stained in Alcian Blue solution (150 mg/L of Alcian Blue, 20% acetic acid, 76% ethanol, 4% water) overnight, and rinsed with 95% ethanol for 3 h to remove excess dye, and the tissues were transparentized with 2% KOH. The bony calcified tissues were then stained with 50 mg/L of alizarin red solution containing 1% KOH. Finally, a 20% glycerol solution containing 1% KOH was used to remove soft tissues.

### 2.3. Micro-CT Analysis

Tibias from embryos were fixed to be analyzed using Scanco medical AG uCT50 (Scanco Medical, Brüttisellen, Switzerland) by JEOL (JEOL Ltd., Tokyo, Japan). The measurement parameters were as follows: voltage, 45 kV; current, 800 kA; resolution, 2 μm; exposure time, 1500 ms. Calcified areas (%) in the trabecular bones were measured using ImageJ. The tibial transverse sections taken with microCT were binarized, and the calcified cancellous bone area and total section area were measured. Calcified area (%) = trabecular bone area/total cross-sectional area. Five identical locations were selected for each group, and means ± standard deviations were calculated.

### 2.4. Primary Cell Culture

Rib cage chondrocytes were isolated and pooled from 9 GF embryos and 9 SPF embryos, as previously described [24,25]. In brief, rib cartilage was collected from fetuses under a microscope and digested with 0.25% trypsin at 37 °C for 5 min to remove soft tissue. The cartilage was then digested with 2 mg/mL of collagenase A (Roche, Basel, Switzerland) at 37 °C for 2 or 3 h to liberate chondrocytes. Harvested chondrocytes were cultured in αMEM + 10% FBS + antibiotics at 37 °C under 5% CO_2_ and used for further analysis. For Alcian Blue staining, cultured cells were washed with PBS and fixed in 95% ethanol. The cells were then stained with 1% Alcian Blue stain containing 0.1 M HCI.

### 2.5. Cell Proliferation

Cell proliferation was quantified using the CellTiter 96 One Solution Cell Assay (MTS; Promega, Madison, WI, USA), according to the manufacturer’s instructions. Primary chondrocytes were seeded on 96-well plates. At 3, 9, and 14 days of culture, cells were washed with PBS and incubated with 80 μL of CellTiter 96 solution. Every hour for 4 h, the absorbance at 490 nm was monitored with a 96-well plate reader (SH1000, Corona, Hitachinaka, Japan), and the absorbance was compared at each time point.

### 2.6. RNA Preparation and RNA Sequence

The RNA preparation was as previously described [21]. In brief, total RNA was isolated from the chondrocyte using Trizol reagent (Invitrogen, Waltham, MA, USA), according to the manufacturer’s instructions. Total RNA was further purified by RNeasy Mini Kit (Qiagen, Hilden, Germany), according to the manufacturer’s instructions. Isolated RNA was quantified by measuring the absorbance at 260 nm, and purity was determined by the 260/280 nm absorbance ratio. The library construction and RNA-seq were performed by DNA Chip Research Inc. (Tokyo, Japan). The NEBNext Ultra II Directional RNA Library Prep kit for Illumina (New England BioLabs Inc., Ipswich, MA, USA) and NEBNext Poly(A) mRNA Magnetic Isolation Module (New England BioLabs Inc., Ipswich, MA, USA) were used for library preparation, according to the manufacturer’s protocol. The libraries of the samples were sequenced using NextSeq500 (Illumina, San Diego, CA, USA) in 75-base-pair (bp) single-end reads. The output raw reads were processed for quality control using FastQC. After read-trimming, the acquired reads were aligned to the reference genome using STAR (Spliced Transcripts Alignment to a Reference), and BAM files were created. The mouse genome UCSC mm10 obtained from Illumina iGenomes (http://jp.support.illumina.com/sequencing/sequencing_software/igenome.html, accessed on 20 September 2019) was used for mapping. To analyze differences in gene expression, k-mean clustering and enrichment analysis were performed using iDEP. 95 [26].

### 2.7. Real-Time PCR

Real-time PCR was performed as previously described [21]. The primer sequences are listed below (Table 1). The mRNA levels were standardized with each mRNA level of Gapdh for each sample [21].

### 2.8. Statistical Analysis

Data were presented as the means ± standard deviation. Student’s unpaired *t*-tests were used to compare GF and SPF mice to determine whether there were any significant differences. All calculations were performed using the statistical software package SPSS 22.0 for Windows (SPSS Japan, Tokyo, Japan). A *p*-value of less than 0.05 was considered statistically significant [21,27].

## 3. Results

### 3.1. Comparison of Fetal and Placental Size from GF and SPF Embryos

**Figure 1:**
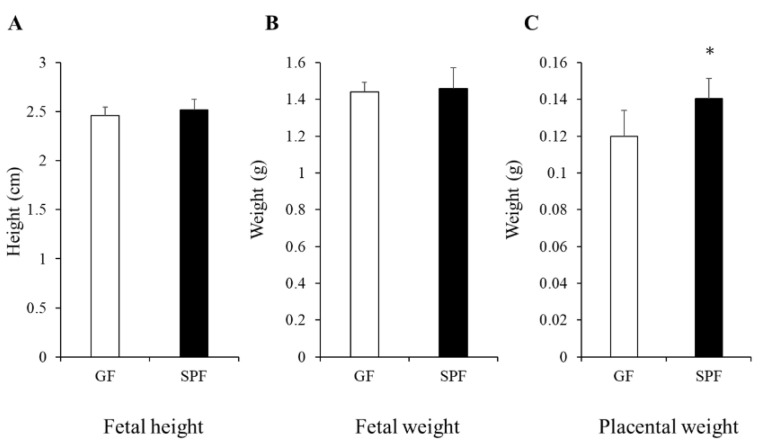
Size of GF and SPF fetus and their placental size. Comparison of body height (**A**) and weight of GF and SPF embryos (**B**). (**A**) The length of the crown-rump length of each fetus were measured. (**B**) Measurement of the weight of each fetus after caesarean section. (**C**) Measuring placental weight after caesarean section. Fetuses at embryonic day 18 (E18) were collected from GF and SPF pregnant mothers. The data are presented as the mean ± SD (*n* = 8, *t*-test, * *p* < 0.05).

To explore the effects of the maternal gut microbiome on fetal physiology, we first compared the fetal physiology and placenta weight. The length of the fetus was measured from the top of the head to the bottom of the rump. Significant differences in fetal head length and body weight were not observed between germ-free (GF) and specific-pathogen-free (SPF) fetuses, whereas the placental weight was significantly greater in SPF mice than in GF mice, indicating more blood supply to the fetus from the SPF uterus of the mother (Figure 1).

### 3.2. Less Mineralization in Fetal SPF Bone

**Figure 2:**
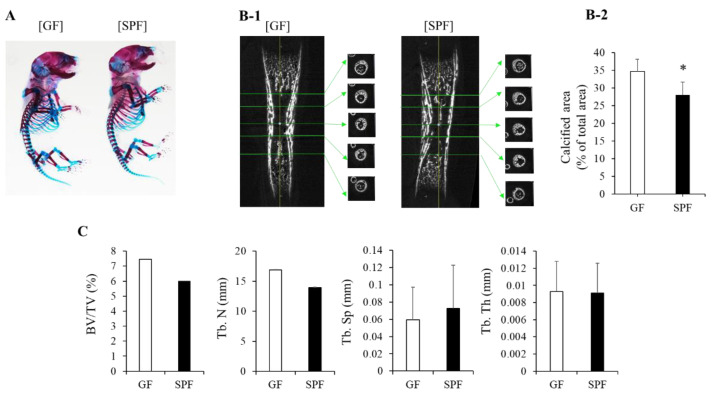
Morphology of GF and SPF embryos. (**A**) Skeletal preparation. (**B-1**) Micro CT analysis of GF and SPF tibiae (E18). Corresponding sagittal and transverse planes were shown. (**B-2**) Average of mineralized area (μm^2^)/total area (μm^2^) from 5 different cross-sections in (**B-1**) is shown. (*t*-test, * *p* < 0.05). (**C**) Mineralized parameters from micro-CT analysis. The ratio of bone volume to total volume (BV/TV) and Tb.N tended to be lower in the SPF group than in GF bones. BV/TV, bone volume/tissue volume; Tb.N, trabecular number; Tb.Sp, trabecular separation; Tb.Th, trabecular thickness.

We reported that the bone mass in SPF mice is lower than that of GF mice in mature mice [18]. In this study, the fetal skeleton was further examined to determine whether differences in the development of the body mass and size were observed at birth or in the fetal period. First, the differences in skeletons between GF and SPF embryos were checked by transparent skeletal preparations, but no significant differences were observed in the calcified bone and cartilage between the two groups (Figure 2A). We also performed a micro-CT analysis of the fetal tibia to monitor the ossification of the fetal bone (Figure 2B,C). The bone volume fraction [bone volume (BV)/total volume (TV)] and trabecular number (Tb.N) tended to be smaller in SPF fetuses compared to GF fetuses (Figure 2C). A comparison of the calcified areas in the cross-sectional views with image analysis showed that the SPF fetuses had smaller calcified areas than the GF fetuses (Figure 2B). On the other hand, bone mass intervals (Tb.Sp) tended to be greater in SPF fetuses than in GF fetuses. Regarding the trabecular thickness (Tb.Th), a similarity was observed between the two groups (Figure 2C). These results may indicate that the maternal gut microbiome affects fetal internal ossification structures, passing through the placenta from the embryonic stage.

### 3.3. Decelerated Chondrocyte Proliferation and Accumulation of Extracellular Matrices in SPF Cartilage

**Figure 3:**
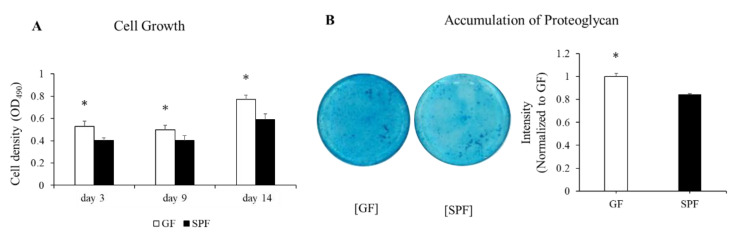
The effects of maternal microbiome on proliferation and accumulation of extracellular matrix in fetal chondrocytes from GF and SPF lib cages. (**A**) Cell proliferation, (**B**) (left) accumulation of Proteoglycan stained with Alcian Blue, (right) staining intensity measured by FIJI/ImageJ. The data are presented as mean ± SD (*n* = 3, *t*-test, * *p* < 0.05). OD = optical density.

Our micro-CT results showed that fetuses in the uterus of the mother mice with the microbiome tended to have less bone mass, indicating decelerated endochondral ossification during longitudinal osteogenesis, at least in part. We therefore decided to isolate and culture primary chondrocytes from the rib cartilage of GF/SPF fetuses. First, we compared the growth rates between SPF and GF embryonic chondrocytes. MTT-based cell-proliferation assays were performed in 3-, 9-, and 14-day cultures. At all culturing timepoints, the cell number was significantly lower in SPF fetus chondrocytes than in GF fetuses (Figure 3A). Cultured chondrocytes from SPF fetuses for 1 month showed less accumulation of the extracellular matrix compared to GF fetuses (Figure 3B). These results indicate that the maternal gut microbiome regulates the proliferation rate and extracellular matrix formation of the fetal chondrocytes.

### 3.4. Gene-Expression Analysis in GF and SPF Fetal Chondrocytes

**Figure 4:**
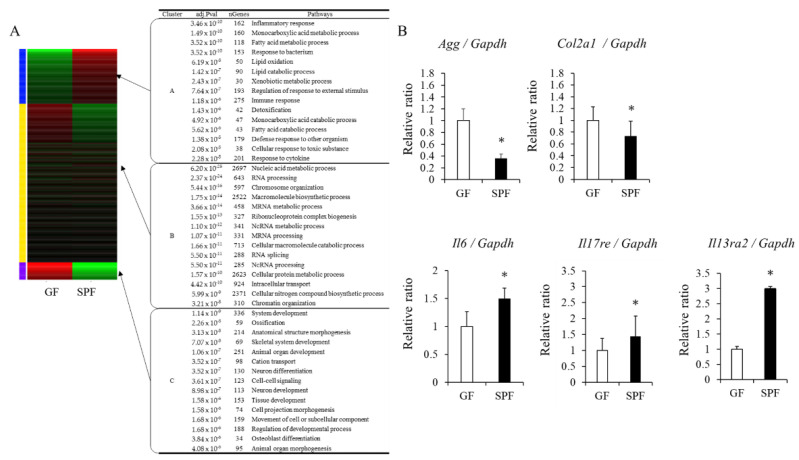
Maternal gut microbiome affects gene expression in chondrocytes of the fetus. (**A**) K-means clustering and enrichment analysis, (**B**) gene expression analysis in primary chondrocytes from GF and SPF rib cages. The data are presented as the mean ± SD (*n* = 3, *t*-test, * *p* < 0.05).

Based on the comparison of the proliferation rate of primary chondrocytes and extracellular matrix formation, we hypothesized that the growth of the chondrocytes was inhibited in the SPF uterus environment. We therefore decided to compare the transcriptome variation in each cell. First, we picked up the gene expression variation by RNA-sequencing analysis, and then we confirmed the gene expression in real-time PCR. K-means clustering showed that genes were differentially expressed between GF and SPF embryo-derived chondrocytes (Figure 4A and the enlarged image in Figure S1), and we focused on two clusters: C. gene pathways related to ossification and growth and development (ossification, skeletal system development, tissue development), which showed higher expression in the GF group; and A. gene pathways related to inflammation (inflammatory response, response to bacteria, immune response, response to cytokines), which showed higher expression in the SPF group. Based on Figure 3B and Figure 4A, genes involved in skeletal development and inflammation were selected, and RT-PCR further confirmed their expression. The expressions of chondrocyte extracellular matrix genes, such as aggrecan (*Agg*) and type II collagen (*Col2a1*) mRNAs, which are involved in skeletal development, were suppressed in SPF fetal chondrocytes compared to GF chondrocytes (Figure 4A,B). This is in line with the results shown in Figure 3B. Furthermore, gene expressions of the pro-inflammatory factors interleukin (IL) 6 (*Il6*), IL17 receptor (*Il17re*), and IL13 receptor 2 (*Il13re2*) mRNAs were significantly enhanced in SPF fetuses’ chondrocytes compared to GF (Figure 4B). These results indicate that the presence of the maternal gut microbiome may be responsible for the expression of inflammatory-related factors in fetal chondrocytes and impair extracellular matrix formation.

## 4. Discussion

The impact of the gut microbiome on our homeostasis seems no longer confined to the individual’s microbiome, as increasing evidence shows that the maternal microbiome affects the growth and metabolism of fetal cells. For the first time, our study shows that the maternal gut microbiome has an inhibitory effect on fetal endochondral bone formation, which suggests that endochondral bone growth is regulated from the start of maternal life.

During the fetal period, the body size and weight of SPF embryos are not significantly different from those of GF embryos, suggesting that the differences in body size due to the presence or absence of a gut microbiome only become apparent after birth. In addition, mineralization parameters (BV/TV and Tb.N) in the skeleton of SPF fetuses tended to be lower than those of GF fetuses. Previous studies have shown that the bone volume of the adult SPF mice was lower than that of GF mice [18,19,21]. Our results point to the fact that the degree of internal ossification is not determined after birth but tends to be present from the start of the prenatal period.

The gut microbiome has further been reported to contribute to the deterioration of cartilage tissue, particularly in osteoarthritis [28]. Genomic comparison of the bacterial flora of the knee joint in healthy subjects and osteoarthritis patients reported that Gram-negative bacteria were detected more frequently in osteoarthritis patients. In SPF primary chondrocytes from fetal cartilage, the proliferation rate of the cells was significantly lower than that of GF fetal-derived chondrocytes, and the accumulation of cartilaginous extracellular matrices was also lower. These results suggest that the growth and maturation of chondrocytes are suppressed in SPF fetuses.

The gut microbiome and immune function are closely linked [29,30,31]. Health conditions during pregnancy have been recently reported to significantly impact immune responses and metabolic functions during the embryonic period and postnatally. Transplantation of the gut microbiome of pregnant mothers with inflammatory bowel disease (IBD) in GF mice has been reported to reduce Treg cells and IgA+ B-cell subsets in the colonic lamina propria [32]. Another study focusing on short-chain fatty acid (SCFA) receptors reported that SCFAs secreted from the gut microbiome of pregnant mice may prevent the development of metabolic syndrome by affecting the postnatal energy homeostasis of the fetus by acting on the fetal short-chain fatty acid receptors [17]. In the present study, the expression of inflammatory factors and receptors was higher in chondrocytes from SPF fetuses than those in GF fetuses. IL-6 and IL-17 were known to inhibit the chondrogenic differentiation of human mesenchymal stem cells [33]. In addition, IL-13Rα2 is a decoy receptor for IL-13, but it has been reported that TNFα and IL-17A synergistically enhance its inflammatory signaling by inducing the expression of IL13Rα2 in fibroblasts [34]. Thus, the presence or absence of maternal intestinal microflora may have a regulatory effect on the cartilage of the fetus through maternal factors. Moreover, such factors could lead to the activation of both osteoclasts and osteoblasts in the presence of the bacterial flora, as we have previously reported [21].

One point is that placental weight was reduced without a gut microbiome. Recent studies on the microbiome of the placenta have been reported, but with conflicting results and no definitive conclusion [35,36]. The placenta provides nourishment for the embryo and protects the embryo from any damage. It is also a place of communication between the fetus and the mother. We previously reported that the estimated fetal growth was suppressed in pregnant women with periodontal disease in late pregnancy compared with healthy controls [37]. As placental nutrient transport is associated with placental weight [38], maternal-gut-microbiota-derived substances may have reached the fetus via the placenta and triggered inflammatory gene expression on the fetal side. Further research is required.

This study focused on the effect of the presence or absence of the maternal gut microbiome on skeletal development on the embryonic side; however, the mechanism of placental function and how the maternal gut microbiome’s condition transferred to the fetus remains unexplored. Further analysis of the potential influence of the maternal gut microbiome on the skeletal development of offspring is also needed.

## 5. Conclusions

The presence of the mother’s gut microbiome influences fetal bone development, including endochondral ossification.

## Figures and Tables

**Table 1:** Primers used for real-time PCR analysis.

Gene	F/R	Primer Sequences (5′–3′)
*Agg*	F	gttcctgcacagcttcacaa
	R	aaacagcccagtgaccattc
*Col2a1*	F	gaactgcaacacattgtggg
	R	attgatggtgaggtgtgcaa
*IL6*	F	agttgccttcttgggactga
	R	cagaattgccattgcacaac
*IL17re*	F	cagtaacagtgacgctagac
	R	acccactagagcggtgagag
*IL13ra2*	F	gcaaaggaggacaaagaggtc
	R	gatttagtgtgctgaaagctctactc
*Gapdh*	F	tgtgatgggtgtgaaccacgagaa
	R	gagcccttccacaatgccaaagtt

## Data Availability

The data of this study are included in the main text of the paper and in the Supplementary Material.

## Supplementary Materials

The following supporting information can be downloaded at: https://www.mdpi.com/article/10.3390/microorganisms10051000/s1, Figure S1: Effect of the presence or absence of maternal gut microbiome on enriched pathways expressed in fetal-derived chondrocytes.
