# Supplementary material for: Maternal Gut Microbiome Decelerates Fetal Endochondral Bone Formation by Inducing Inflammatory Reaction
Source: Microorganisms. 2022 May 10;10(5):1000. doi: 10.3390/microorganisms10051000 (PMC9147398; doi:10.3390/microorganisms10051000)
Supplement: Supplementary file 1 [file microorganisms-10-01000-s001.zip › microorganisms-1681268-supplementary.pdf]

Supplementary figure

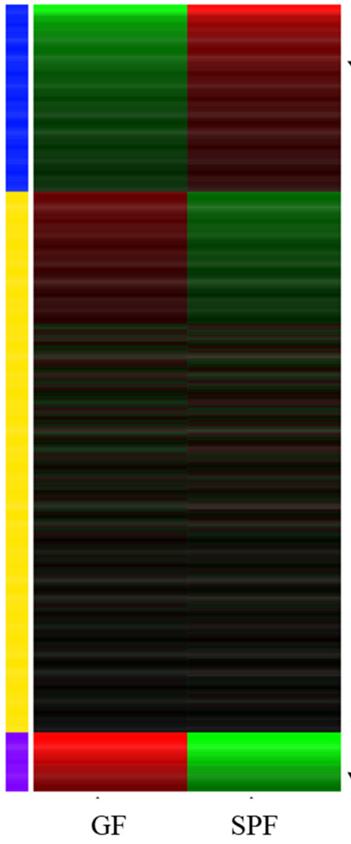

Cluster	adj.Pval	nGenes	Pathways
	3.46×10^{-10}	162	**Inflammatory response**
	1.49×10^{-10}	160	Monocarboxylic acid metabolic process
	3.52×10^{-10}	118	Fatty acid metabolic process
	3.52×10^{-10}	153	Response to bacterium
	6.19×10^{-8}	50	Lipid oxidation
	1.42×10^{-7}	90	Lipid catabolic process
	2.43×10^{-7}	30	Xenobiotic metabolic process
A	7.64×10^{-7}	193	Regulation of response to external stimulus
	1.18×10^{-6}	275	**Immune response**
	1.43×10^{-6}	42	Detoxification
	4.92×10^{-6}	47	Monocarboxylic acid catabolic process
	5.62×10^{-6}	43	Fatty acid catabolic process
	1.38×10^{-5}	179	Defense response to other organism
	2.08×10^{-5}	38	Cellular response to toxic substance
	2.28×10^{-5}	201	**Response to cytokine**
	6.20×10^{-25}	2697	Nucleic acid metabolic process
	2.37×10^{-24}	643	RNA processing
	5.44×10^{-16}	597	Chromosome organization
	1.75×10^{-14}	2522	Macromolecule biosynthetic process
	3.66×10^{-14}	458	MRNA metabolic process
	1.55×10^{-13}	327	Ribonucleoprotein complex biogenesis
	1.10×10^{-12}	341	NcRNA metabolic process
B	1.07×10^{-11}	331	MRNA processing
	1.66×10^{-11}	713	Cellular macromolecule catabolic process
	5.50×10^{-11}	288	RNA splicing
	5.50×10^{-11}	285	NcRNA processing
	1.57×10^{-10}	2623	Cellular protein metabolic process
	4.42×10^{-10}	924	Intracellular transport
	5.99×10^{-9}	2371	Cellular nitrogen compound biosynthetic process
	3.21×10^{-8}	310	Chromatin organization
	1.14×10^{-9}	336	System development
	2.26×10^{-8}	59	**Ossification**
	3.13×10^{-8}	214	Anatomical structure morphogenesis
	7.07×10^{-8}	69	**Skeletal system development**
	1.06×10^{-7}	251	Animal organ development
	3.52×10^{-7}	98	Cation transport
	3.52×10^{-7}	130	Neuron differentiation
C	3.61×10^{-7}	123	Cell-cell signaling
	8.98×10^{-7}	113	Neuron development
	1.58×10^{-6}	153	**Tissue development**
	1.58×10^{-6}	74	Cell projection morphogenesis
	1.68×10^{-6}	159	Movement of cell or subcellular component
	1.68×10^{-6}	188	Regulation of developmental process
	3.84×10^{-6}	34	Osteoblast differentiation
	4.08×10^{-6}	95	Animal organ morphogenesis

Supplementary Figure S1

Effect of the presence or absence of maternal gut microbiome on enriched pathways expressed in fetal-derived chondrocytes.

Enriched pathways in GF-derived and SPF-derived chondrocytes from k-means clusters were annotated by iDEP web application (integrated Differential Expression and Pathway analysis, http://bioinformatics.sdstate.edu/idep95/) using gene ontology annotation (GO biological process). Pathways marked in yellow lines were further investigated using quantitative RT-PCR (see text).
